# Quality Control and Mating Performance of Irradiated *Glossina palpalis gambiensis* Males

**DOI:** 10.3390/insects13050476

**Published:** 2022-05-19

**Authors:** Kadidiata Ilboudo, Karifa Camara, Ernest W. Salou, Geoffrey Gimonneau

**Affiliations:** 1 Centre International de Recherche—Développement sur l’Elevage en zone Subhumide, Bobo-Dioulasso 01 BP 454, Burkina Faso; kadidia124@gmail.com (K.I.); camarakariffa@gmail.com (K.C.); geoffrey.gimonneau@cirad.fr (G.G.); 2 Institut Supérieur des Sciences et de Médecine Vétérinaire (ISSMV), Dalaba BP 09, Guinea; 3 Département de Sciences Biologiques/UFR-SVT, Université Nazi Boni (UNB), Bobo-Dioulasso BP 1091, Burkina Faso; 4 Centre de coopération Internationale en Recherche Agronomique pour le Développement (CIRAD), Unité Mixte de Recherche, Interactions Hôtes-Vecteurs-Parasites-Environnement dans les Maladies Tropicales Négligées Dues aux Trypanosomatidés (UMR INTERTRYP), F-34398 Montpellier, France; 5 Interactions Hôtes-Vecteurs-Parasites-Environnement dans les Maladies Tropicales Négligées dues aux Trypanosomatides (INTERTRYP), Université de Montpellier, Centre de coopération Internationale en Recherche Agronomique pour le Développement (CIRAD), Institut de Recherche pour le Développement (IRD), F-34398 Montpellier, France

**Keywords:** sterile insect technique, tsetse fly, sterility rate, competitiveness

## Abstract

**Simple Summary:**

In vector control programs based on the sterile insect technique (SIT), the biological quality of sterile males must be as high as possible to compete with their wild counterparts. This study evaluates the biological quality and mating performance of sterile male *Glossina palpalis gambiensis* produced in a mass-rearing facility in Burkina Faso. It shows that irradiation had no effect on the adult emergence rate but reduced the percentage of operational flies and male fly survival. Irradiation had no effect on mating performances, as all females were inseminated, and the sterile males competed well with unirradiated males for virgin females in walk-in field cages. However, the sterility rate induced in females was lower than expected (89.67%). This study indicates that, under experimental field cage conditions, the *G. p. gambiensis* males produced at the CIRDES are well-suited for use in area-wide integrated pest management (AW-IPM) programs that have an SIT component. The reduction in the sterility rate highlights the importance of regularly monitoring the biological parameters of sterile males and the radiation source.

**Abstract:**

The biological quality of sterile male insects produced in a mass-rearing facility is a prerequisite for the success of the SIT, which is a component of area-wide integrated pest management (AW-IPM). Indeed, sterile male insects released in the field must have a good mating performance in order to compete with wild males, but they must also present the required level of sterility. In the present study, the biological quality of sterile male *Glossina palpalis gambiensis* produced in a mass-rearing insectary was assessed through quality control testing. The mating performance of irradiated males was assessed in walk-in field cages. Irradiation had no effect on adult emergence but significantly reduced the percentage of operational flies (from 89.58% to 79.87%) and male survival (from 5 to 4 days, on average). However, irradiation did not impact the sterile male insemination potential, with all females inseminated and more than 80% of the spermathecae completely filled. The rate of induced sterility in females was 89.67% due to a dose rate decrease of the radiation source. Moreover, sterile males were able to compete successfully with untreated fertile males for untreated females in walk-in field cages. This study confirmed that the flies were still competitive and stressed the importance of regularly checking the radiation source parameters.

## 1. Introduction

Tsetse flies (Diptera: Glossinidae) are the cyclical vectors of trypanosomes, the causative agents of African animal trypanosomosis (AAT) or nagana in animals and human African trypanosomosis (HAT) or sleeping sickness in humans [1]. Through their distribution, tsetse flies impair the exploitation of fertile lands, covering more than ten million km^2^ in sub-Saharan Africa [2]. The presence of tsetse flies and the disease they transmit leads to economic losses in livestock and crop production estimated at USD 4.75 billion per year [3]. There is no vaccine against AAT, and chemotherapy-based management strategies have shown limitations due to the development of parasite resistance to the available trypanocidal drugs [4,5]. In such a context, vector management strategies following area-wide integrated pest management (AW-IPM) approaches [6,7] are the most efficient and sustainable options to manage the disease.

Given an AW-IPM approach, the management of tsetse fly populations is achieved through four economically and environment-friendly methods. Three methods are insecticide-based: the sequential aerosol technique (SAT), the deployment of insecticide-impregnated traps/targets and live bait technology. The fourth method is the autocidal sterile insect technique (SIT) [7,8]. The SIT is based on the sterilizing effect of radiation on insects [9]. Males of the target species are mass-reared, sterilized by radiation and released in the field to compete with their wild counterparts for females. A mating of a sterile male with a wild female results in no offspring and will lead to population reduction or elimination [9]. Several programs have used this technique to successfully control, suppress or eradicate species of veterinary and agricultural importance, such as fruit flies [10], moths [11], screwworm flies [12] and tsetse flies [13].

In 2005, the Senegalese Government embarked on an AW-IPM project that included an SIT component to eradicate *G. palpalis gambiensis* from the Niayes area in Western Senegal [14]. Sterile males were supplied as irradiated pupae by four production centers, two in Burkina Faso and one each in Slovakia and Austria. The insectary of the Centre International de Recherche-Développement sur l’Elevage en zones Subhumides (CIRDES) in Burkina Faso has maintained a *G. p. gambiensis* (BKF strain) colony since the 1970s that was used in an eradication program in the Sidéradougou area of Burkina Faso in the 1980s [15]. Before initiating the release of sterile males in the target area, studies were performed to determine the optimal radiation dose that induces a high level of sterility but with a minimal impact on the male competitiveness [16,17,18]. Indeed, one of the main challenges of the SIT is to find the best balance between the radiation dose and male competitiveness [9]. A radiation dose of up to 110 Gy had no visible effect on the mating behavior, insemination rate, sperm motility and competitiveness of *G. p. gambiensis* [17]. 

The success of SIT programs strongly depends on the biological quality of sterile males, which must be able to compete with local wild males [19]. Therefore, a regular evaluation of the sterile male quality and competitiveness must be performed to maximize the success of eradication programs. The last published studies on the *G. p. gambiensis* BKF strain showed it was still competitive, even though this strain was established 43 years ago [20,21,22]. Taking a quality control approach, this study assessed the biological quality and mating performance of sterilized male *G. p. gambiensis*.

## 2. Material and Methods

### 2.1. Tsetse Flies

All the experiments were carried out with *G. p. gambiensis* from a laboratory colony established in 1975 (known as the BKF strain) at the Centre International de Recherche-Développement sur l’Elevage en zones Subhumides (CIRDES), Burkina Faso [23]. Flies were maintained under controlled environmental conditions (temperature 25 ± 1 °C, 75 ± 5% relative humidity and 12:12 h light:dark) and were daily offered irradiated bovine blood for feeding following the standard rearing procedures using an in vitro silicon membrane feeding system [24]. The blood originated from the local abattoir in Bobo-Dioulasso, with which CIRDES has a long-term collaboration agreement for blood collection to feed tsetse fly colonies. The experiments described below were performed between January and June 2018.

### 2.2. Chilling and Irradiation of Male Pupae and Dosimetry of the Irradiator

After most of the female flies emerged, the remaining male *G. p. gambiensis* pupae were chilled at 8–10 °C for 24 h to lower their metabolic rate and to prevent emergence [25,26]. The pupae were irradiated under chilled (8–10 °C) conditions in a ^137^Cs source for 24 min 30 s to give a dose of 110 Gy. Males used for the control treatments were chilled during the same period but not irradiated.

Based on the sterility rate of the males obtained in this study, a dosimetry evaluation of the radiation source was performed in 2021. The CIRDES irradiator was a ^137^Cs gamma source installed in 1973. It reached its half-life in 2003, and the last dosimetry performed before our study was done in 2012, showing that 24 min 30 s was necessary to achieve a target-absorbed dose of 110 Gy. Although it was not possible to update the dosimetry information before our study due to the lack of dosimeters, it was performed in 2021. This allowed us to determine the dose to which our flies were exposed afterward. A Radcal^®^ ionization chamber (reference 10 × 5–0.6, serial number 9407, Mondrovia, CA, USA), previously calibrated, was used to assess the dose rate and absorbed dose.

### 2.3. Experiment 1: Quality Control of Irradiated Male Pupae

The quality control of male pupae was based on the methodology proposed by Seck et al. [27]. This procedure evaluates the quality of sterile males measured as the percentage of operational flies corresponding to the percentage of flies escaping a flight device. Briefly, pupae were placed in Petri dishes and covered with ~1 cm of autoclaved sand mixed with a fluorescent dye (DayGlo^®^, Cleveland, OH, USA) (0.5 g dye/200 g of sand) to mimic natural emergence conditions and to allow the marking of the emerged flies to discriminate the trapped sterile male flies from wild flies during entomological monitoring in AW-IPM programs that have an SIT component. A flight cylinder, i.e., a PVC tube 10 cm high and 8.4 cm in diameter, was placed on the Petri dishes with the inner walls coated with unscented talcum powder to prevent flies from crawling out. The flies that flew out the flight cylinder were considered ‘operational flies’. Flies with deformed wings and those with normal wings but unable to escape the flight tube were counted and classed as non-flyers, as well as the number of pupae that did not emerge.

Survival of the sterile males that escaped the flight cylinder was assessed under stress conditions (no blood meal). Every morning, newly emerged flies were collected and transferred to standard fly holding cages. All flies that emerged on a given day were pooled in one cage. Dead flies were counted daily and removed from the cages.

The experiment was replicated 18 times, with each replicate corresponding to three cages of every 50 irradiated pupae and one control cage with 50 non-irradiated pupae. The pupae used in each replicate were from different larviposition dates.

### 2.4. Experiment 2: Insemination Capacity of Irradiated Male G. p. gambiensis

Males were chilled and irradiated as previously described [25,26]. Virgin females were collected as they emerged from the pupae and held in separated cages. Three experimental cages (37 cm in length × 6 cm in height × 19 cm in width) with 10 six-day-old sterile males and 30 three-day-old virgin females (i.e., a ratio of one male to three females) were set up at the same time. Simultaneously, three control cages were set up with non-irradiated males of the same male:female ratio of 1:3. Flies were offered a blood meal daily and maintained under standard rearing conditions for 12 days. The experiment was replicated five times. The replicate corresponded to three control cages and three cages with irradiated males. Adults from a different insectary batch were used for each replicate. At the end of the experiment, all female flies were dissected to assess insemination and the level of spermathecae fill. Dissections were carried out in a drop of physiological solution (lactated ringer‘s solution) under a binocular microscope [28]. The spermathecal value was obtained by assessing the content of the two spermathecae and adding the values. The spermathecae were classed as empty, 0; quarter-full, 0.25; half-full, 0.5, three-quarters full, 0.75 and full, 1 [29].

### 2.5. Experiment 3: Female-Induced Sterility Levels when Mated with Irradiated Male Glossina palpalis gambiensis

The experimental procedure was similar to experiment 2. Three experimental cages with 10 six-day-old sterile males and 30 three-day-old virgin females (i.e., a ratio of one male to three females) were set up at the same time. Simultaneously, three control cages were set up with non-irradiated males. Flies were fed daily and maintained under standard rearing conditions. Here, the experiment ended after the first larviposition cycle, i.e., five days after the first pupae was observed. The number of pupae produced and female mortality were recorded. The experiment was replicated five times. A replicate corresponded to three control cages and three cages with irradiated males. Adults from different insectary batches were used for each replicate.

Female-induced sterility was assessed for the first ovulation cycle, i.e., from the first day of larviposition to five days after. The induced sterility rate was calculated according to Taze et al. [17]. For each treatment and replicate, the number of pupae produced and the number of reproductive females per day were calculated. The number of reproductive females per day corresponded to the sum of live females in the cage from the first day of larviposition until the end of the follow-up (i.e., day five). Productivity was calculated as follows (1):

(1) $$P=\frac{\mathrm{pupae}\;\mathrm{number}}{\mathrm{number}\;\mathrm{of}\;\mathrm{breeding}\;\mathrm{females}/\mathrm{day}}.$$

Considering that females of the control treatment had a sterility rate of 0, the sterility rate of the females from the irradiated treatment was deduced [17].

### 2.6. Experiment 4: Male Mating Performance in a Walk-In Field Cage 

Males were chilled and irradiated as previously described. Sterile and fertile males emerged through autoclaved sand mixed with a fluorescent dye (DayGlo^®^) (0.5 g dye/200 g of sand) of different colors (green and red, respectively) to allow discrimination after the mating experiment. Virgin females were collected as they emerged from the pupae and held in separated cages. Males and females were kept under standard insectary conditions until they reached sexual maturity, i.e., six days for males and three days for females, and they were offered a blood meal daily, except on the day of the experiment.

The mating performance of the irradiated male flies was assessed in walk-in field cages in June 2018 [20,29,30,31]. The experiment took place in a stable under mosquito netting in a release arena of the following dimensions (2.65 m (length) × 2.2 m (width) × 2 m (height). Two citrus plants (*Citrus sinensis* L., one meter in height) were placed in the center of the cage [20,29]. During the experiment, the temperature and relative humidity were recorded every 30 min using a Hobo data logger.

The experiments started at 9:00 a.m. and ended at 12.00 a.m. Sixty virgin males, including thirty irradiated males and thirty non-irradiated males, competed for mating with 30 virgin females. The females were released first, followed by the males five minutes later in the middle of the cage. The observer was in the field cage for the 3-h duration of the experiment. Mating pairs were collected in individual vials, and the time was recorded (start of mating and mating duration). The experiment was replicated five times. At the end of each experiment, mated females were collected, immobilized at −5 °C and dissected to check spermathecal fill. Mating flies (males) were killed by chilling (20 min at −20 °C) and identified using a fluorescence camera (Dino-lite, Taipei, Taiwan) to observe the fluorescent powder color fixed in the ptilinum. Flies (males and females) that did not mate were collected and killed.

### 2.7. Mating Indices and Data Analysis

Several mating competitiveness indicators were used to assess the male performance: the propensity of mating (PM), relative mating index (RMI) and relative mating performance (RMP). The propensity of mating (PM) was defined as the overall proportion of released females that mated. The RMI was defined as the number of pairs of one treatment group as a proportion of the total number of mating [29]. An RMI value of 0.5 showed an equal competitiveness between the strains. The RMP was defined as the difference between the number of matings of two treatments of males as a proportion of the total number of matings [29]. This index is a measure of the mating compatibility. In addition, the mating latency time, mating duration, insemination rate and the spermathecal fill of each mated female were determined.

Data from the quality control of the irradiated male pupae experiment were analyzed using a binomial generalized linear mixed-effect model (package lme4). The emergence rate, percentage of flyers, flies with normal wings but unable to fly and flies with deformed wings were set as the response variables, the treatment (control or irradiated males) as the fixed effect and the replicate as a random effect. The male insemination capacity was analyzed, again using a linear mixed-effect model (package lme4). The spermathecal fill was set as the response variable, the treatment as the fixed effect and the replicate as a random effect.

The survival of flies kept under starvation was analyzed using Kaplan–Meier survival curves. The survival curves were compared using the ‘coxme’ function, where irradiation treatment was used as the explanatory variable, survival as the response variable and replicates were used as random effects.

Female productivity mated with sterile males was analyzed using a generalized linear mixed-effect model. Female productivity was set as the response variable, the treatment as the fixed effect and the replicate as a random effect.

The mating competitiveness indicator from the walk-in field cage experiment was analyzed using a linear mixed-effect model to compare male competitiveness parameters (i.e., PM, RMI and RMP) and the spermathecal value between treatments (control or irradiated males). The mating latency and mating duration were analyzed using a linear mixed-effect model with the mating latency and mating duration set as the response variables, the treatment as the fixed effect and the replicate as a random effect. R software (version 3.6.0) was used for all statistical analyses [32].

## 3. Results

### 3.1. Experiment 1: Quality Control of Irradiated Male Pupae

The mean emergence rate of the untreated flies was not significantly different from the irradiated flies (likelihood-ratio test: χ^2^ = 2.992, df = 1, *p* = 0.083; Table 1). The percentage of operational flies was significantly higher for the control flies than for flies of the irradiated treatment group (likelihood-ratio test: χ^2^ = 43.205, df = 1, *p* < 0.001; Table 1). The proportion of flies with normal wings that were not capable of escaping from the flight cylinder was significantly lower in the control group than in the irradiation treatment group (likelihood-ratio test: χ^2^ = 43.205, df = 1, *p* < 0.001; Table 1). The percentage of flies with deformed wings was lower in the control group as compared with the irradiated treatment group (likelihood-ratio test: χ^2^ = 8.677, df = 1, *p* = 0.003; Table 1).

**Figure 1:**
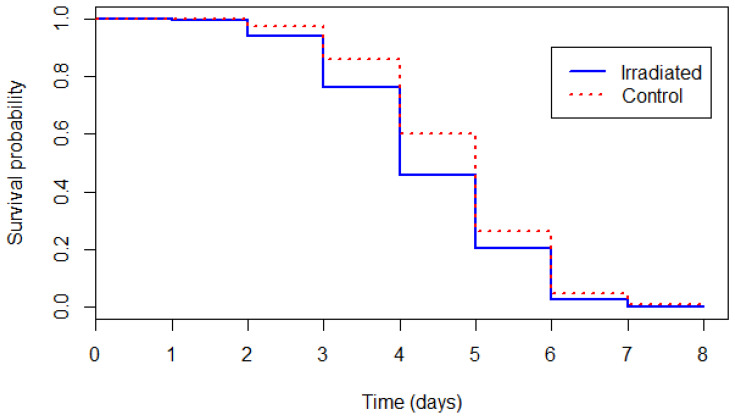
Survival curves of the control and irradiated male tsetse flies under starvation stress conditions.

The survival curves of untreated and irradiated flies kept under starvation are presented in Figure 1. Flies from the control group survived significantly longer than flies from the irradiated pupae group (likelihood-ratio test: χ^2^ = 32.814, df = 1, *p* < 0.001). The mean survival was 4.75 days ± 1.17 for untreated flies and 4.39 days ± 1.22 for the flies that emerged from irradiated pupae.

### 3.2. Experiment 2: Sterile Male Insemination Capacity

Dissection of females after being confined with males for 12 days showed that 100% were inseminated in both the untreated control and the irradiated treatment groups. The insemination levels were similar between the control and treatment groups, with more than 80% of the spermathecae completely filled (Table 2). The spermathecae fill was similar between the control and the treatment groups for the levels of one-quarter (1/4) (likelihood-ratio test: χ^2^ = 0.228, df = 1 *p* = 0.63), three-quarters (3/4) (likelihood-ratio test: χ^2^ = 0, df = 1, *p* = 1.00) and full (1) (likelihood-ratio test: χ^2^ = 1.427, df = 1, *p* = 0.23). Only in the half (1/2)-filled level were significantly more females inseminated by irradiated males (likelihood-ratio test: χ^2^ = 5.377, df = 1, *p* = 0.02).

### 3.3. Experiment 3: Female Induced Sterility Levels Mated with Irradiated Male Glossina palpalis gambiensis

Of the 450 females mated with non-irradiated males, 381 (84.67%) females survived up to day 18 after emergence compared to 344 (76.44%) females that mated with irradiated males. Female productivity was significantly higher for females mated with the untreated control males than for those mated with irradiated sterile males (likelihood-ratio test: χ^2^ = 136.17, df = 1, *p* < 0.001) with 284 and 26 pupae produced, respectively (Table 3). The rate of induced sterility in females mated with irradiated males was 89.7% (Table 3).

### 3.4. Experiment 4: Male Mating Performance in Walk-In Field Cage

During this experiment, the temperature ranged between 25 and 29 °C and the relative humidity between 50 and 94% in the field cage. The mating latency and mating duration were similar for the matings with irradiated and non-irradiated males (likelihood-ratio test: χ^2^ = 0.079, df = 1, *p* = 0.778 for mating latency and likelihood-ratio test: χ^2^ = 0.385, df = 1, *p* = 0.537 for mating duration) (Table 4).

A propensity of mating (PM) of 0.57 was obtained, and the majority of the mated females dissected were inseminated (Table 4). The average relative mating index (RMI) was not significantly different (likelihood-ratio test: χ^2^ = 3.384, df = 1, *p* = 0.103) for irradiated (0.44 ± 0.1) and non-irradiated males (0.56 ± 0.1).

The relative mating performance (RMP) of 0.07 indicated that the mating performance of untreated males and those irradiated as pupae was almost equal.

The spermathecae fill was similar between females mated with untreated males and irradiated males for the four levels: empty (0) (likelihood-ratio test: χ^2^ = 0.783, df = 1, *p* = 0.376), one-quarter (1/4) (likelihood-ratio test: χ^2^ = 1.6, df = 1, *p* = 0.205), half (1/2) (likelihood-ratio test: χ^2^ = 0.4, df = 1, *p* = 0.527) and full (1) (likelihood-ratio test: χ^2^ = 0.287, df = 1, *p* = 0.591). Only in the three-quarters-level (3/4) spermathecal fill group (likelihood-ratio test: χ^2^ = 7.598, df = 1, *p* = 0.006) were females significantly more inseminated by untreated males. The insemination rate was 89.58% and 78.75% for females mated with non-irradiated and irradiated males, respectively (Table 4).

## 4. Discussion

In all the AW-IPM programs that include an SIT component, the biological quality and competitiveness of sterile males are important parameters. Indeed, the success of the SIT is based on the ability of released sterile males to compete with wild males for wild females. Therefore, quality control of the key parameters, such as the sterility rate, longevity and competitiveness of sterile males, must be assessed regularly.

The experiment on the quality control of irradiated pupae showed that irradiation had no effect on adult fly emergence (94.6 ± 5.93% and 92.9 ± 5.79% for the control and irradiated pupae, respectively). Similar results were obtained in previous studies, and no differences were observed in the emergence rates between chilled irradiated and non-irradiated pupae from the same tsetse flies colony [26,33] but, also, for *G. tachinoides* pupae irradiated with 120 Gy on day 28 post-larviposition [34]. However, irradiation reduced the percentage of operational flies by 9.7% (from 89.6% for the control flies to 79.87% for the irradiated flies). This reduction must be due to irradiation, as it was the only factor that was different between the control and the treatment group. Although this reduction was expected since ionizing radiations are known to affect insect performance through somatic cell damage [35], it was much lower than the 20% loss of operational flies observed after chilling and irradiation by Diallo et al. [33]. The survival of flies under starvation was also significantly affected by irradiation. Here again, this result was in line with a previous work that showed that irradiation affects the survival of irradiated flies [17] but contrasted with a recent study performed on the same tsetse fly colony [33], where the authors observed no effect of irradiation on fly survival under starvation. These differences between studies may be due to other factors, such as blood diet quality and female performance at the time of these studies. Indeed, the survival of progeny mainly depends on the female feeding quality during larval development, which determines the level of fat reserves in future imagoes [1]. These results on the biological quality of sterile males after irradiation were in line with expectations for a SIT program, with high emergence and operational fly rates, and a longevity sufficient to allow males to find wild females [19].

In addition to good biological quality, sterile males must be able to compete with wild counterparts for females and induce a significant level of sterility in females. The sterile male insemination capacity and female-induced sterility levels were assessed under insectary conditions. The insemination capacity was similar between irradiated and non-irradiated males. The rate of inseminated females was 100%, and the spermathecal fill was full for more than 80% of the females. This result was similar to previous studies showing that the irradiation of *G. p. gambiensis* at a dose of 110 Gy did not affect the male insemination capacity [17,21] and has also been observed in other species such as *G. f. fuscipes* [36] and *G. brevipalpis* [31].

The mating of irradiated males induced 89.7% sterility in the untreated females. This sterility level was lower than the 97.7% sterility obtained by Taze et al. [17], with flies of the same species and colony but irradiated as adults. It was also lower than other tsetse species from other mass-rearing insectaries, which range between 95 and 100% sterility [31,37,38,39] for the recommended irradiation dose. According to this result, a dosimetry study was performed in 2021 to update the irradiator dose rate. Between 2012 and 2021, the dose rate decreased by 1.785 Gy/min. Based on this data, it could be estimated that flies exposed in 2018 to radiation received a dose of 81 Gy instead of the 110 Gy expected. This reduction in the total dose and dose rate explained the 89.7% sterility observed. It also explained the 20% difference observed in the rate of operational flies between this study and Diallo et al. [33].

This result stressed the importance of regularly controlling the dosimetry parameters of radioactive sources in order to reach the targeted total dose and ensure adequate sterility and insect competitiveness [9]. In this line, a recent work of Yamada et al. [40] showed that the total dose should not be considered as the main parameter in sterilizing insects. The authors highlighted in irradiated mosquitoes a nonlinear interaction between the dose and dose rate that may change with the dose. As a result, these findings suggest that the irradiation dose currently used to sterilized tsetse flies could be optimized according to the dose rate. This parameter was not taken into account in past studies [17,31,41,42,43] and the fine tuning of the dose and dose rate and could lead to more competitive sterile males.

The results from the walk-in field cages indicate that irradiated *G. p. gambiensis* males are still as competitive as non-irradiated males. Indeed, our results showed that irradiated males that emerged from the pupae that were cooled at 8 ± 2 °C for 24 h formed mating pairs sooner than males that were not irradiated. An increase in the mating latency will result in a loss of competitiveness in the field, as wild males will mate quicker than sterile ones. Moreover, the mating duration was similar between irradiated and non-irradiated males, and the majority of mated females were inseminated. This was in accordance with previous walk-in field cage evaluations [20,26] and, also, field releases in Burkina Faso, where studies have shown that the competitiveness of the irradiated males did not differ from the untreated males [21]. In addition, the propensity for mating was 0.57, highlighting that the environmental conditions in the field cage were adequate for this evaluation and that the observer’s presence had only minimal interference on the mating behavior of the flies. This index was close to the index of 0.64 obtained by Mutika et al. [20], who performed the last study on the mating performance of the *G. p. gambiensis* BKF strain in 2002. Similar results were found for other tsetse species, such as *G. brevipalpis* [31] and *G. p. palpalis* [36].

## 5. Conclusions

In this study, the male *G. p. gambiensis* flies from the CIRDES colony showed good biological parameters after irradiation and remained competitive in the semi-field experiment after almost 43 years of colonization. However, the sterility rate induced in females was lower than expected due to late update of the dosimetry parameters and reinforced the importance of regularly monitor the irradiation parameters, male biological quality and mating performances in insect mass-rearing facilities.

## Figures and Tables

**Table 1:** Average values ± SD (%) of different parameters of the quality control by treatment.

Treatment	Emergence Rate	Flight Ability	Non-Fliers Flies	Flies with Deformed Wings
Control	94.55 ± 5.93 ^a^	89.58 ± 13.29 ^a^	7.83 ± 9.95 ^a^	2.59 ± 4.38 ^a^
Irradiated	92.92 ± 5.79 ^a^	79.87 ± 12.60 ^b^	15.08 ± 10.42 ^b^	5.05 ± 3.57 ^b^

Values with the same letters (amongst columns) are not significantly different (*p* > 0.05).

**Table 2:** Reproductive status of *Glossina palpalis gambiensis* females mated with irradiated and non-irradiated males dissected after 12 days of mating.

Treatment	No. Females Dissected	Insemination %	Spermathecae Fill (%)			
			0.25	0.5	0.75	1
Control	368	100	18 (4.89)	16 (4.35)	35 (9.51)	299 (81.25)
Irradiated	406	100	14 (3.45)	27 (6.65)	35 (8.62)	330 (81.28)

**Table 3:** Effect of irradiation on the fertility of irradiated male tsetse flies.

Treatment	No. Pupae Produced	No. Female X Days	Productivity per Female per Days	Sterility %
Control	284	1821	0.1559	0
Irradiated	26	1608	0.0161	89.7

**Table 4:** Summary mating parameters of *Glossina palpalis gambiensis* irradiated and non-irradiated males mated with virgin females.

							Spermathecae Fill						
Treatments	Possible Pairs	Pairs	Mating Latency (Min ± SD)	Mating Duration (Min ± SD)	Propensity of Mating (PM)	Relative Mating Index (RMI ± SD)	0 (0%)	0.25 (%)	0.5 (%)	0.75 (%)	1 (%)	Insemination Rate (%)	Relative Mating Performance (RMP)
Control	150	48	83.9 ± 48.83	45.48 ± 12.50	0.57	0.56	5 (10.42)	4 (8.33)	1 (2.08)	7 (14.58)	31 (64.58)	89.58	0.07
Irradiated	-	38	81.9 ± 54.79	45.16 ± 15.62	-	0.44	8 (21,05)	2 (5.26)	2 (5.26)	0	26 (68.42)	78.75	

## Data Availability

The data that support the findings of this study are openly available in Dataverse at https://doi.org/10.18167/DVN1/DFHSYT (accessed on 16 May 2022).
